# Ruthenium(η^6^,η^1^-arene-CH_2_-NHC) Catalysts for Direct Arylation of 2-Phenylpyridine with (Hetero)Aryl Chlorides in Water

**DOI:** 10.3390/molecules23030647

**Published:** 2018-03-13

**Authors:** Nazan Kaloğlu, İsmail Özdemir, Nevin Gürbüz, Hakan Arslan, Pierre H. Dixneuf

**Affiliations:** 1 Department of Chemistry, Faculty of Science and Arts, İnönü University, 44280 Malatya, Turkey; nzntemelli@hotmail.com (N.K.); nevin.gurbuz@inonu.edu.tr (N.G.); 2 Catalysis Research and Application Center, İnönü University, 44280 Malatya, Turkey; 3 Faculty of Arts and Science, Department of Chemistry, Mersin University, 33343 Mersin, Turkey; hakan.arslan@mersin.edu.tr; 4 Institut des Sciences Chimiques de Rennes, Université de Rennes 1, 35042 Rennes, France; pierre.dixneuf@univ-rennes1.fr

**Keywords:** homogeneous catalysis, ruthenium, *N*-heterocyclic carbene, 2-phenylpyridine, direct arylation, single crystal structure

## Abstract

A series of new benzimidazolium halides were synthesized in good yields as unsymmetrical *N*-heterocyclic carbene (NHC) precursors containing the N–CH_2_–arene group. The benzimidazolium halides were readily converted into ruthenium(II)–NHC complexes with the general formula [RuCl_2_(η^6^,η^1^–arene–CH_2_–NHC)]. The structures of all new compounds were characterized by ^1^H NMR (Nuclear Magnetic Resonance), ^13^C NMR, FT-IR (Fourier Transform Infrared) spectroscopy and elemental analysis techniques. The single crystal structure of one benzimidazole ruthenium complex, **2b**, was determined. The complex is best thought of as containing an octahedrally coordinated Ru center with the arene residue occupying three sites, the remaining sites being occupied by a (carbene)C–Ru bond and two Ru–Cl bonds. The catalytic activity of [RuCl_2_(η^6^,η**^1^**–arene–CH_2_–NHC)] complexes was evaluated in the direct (hetero)arylation of 2-phenylpyridine with (hetero)aryl chlorides in water as the nontoxic reaction medium. These results show that catalysts **2a** and **2b** were the best for monoarylation with simple phenyl and tolyl chlorides. For functional aryl chlorides, **2d**, **2e**, and **2c** appeared to be the most efficient.

## 1. Introduction

**Figure 1:**
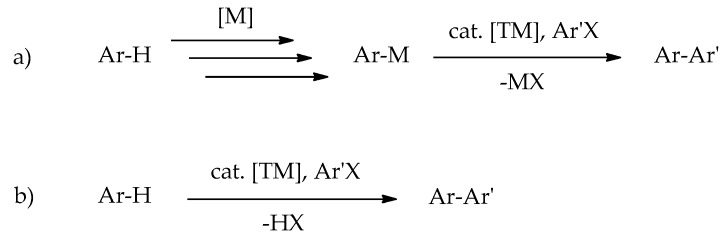
Coupling reactions: (**a**) Traditional cross-coupling; and (**b**) direct arylation.

The prevalence and importance of biaryl compounds in natural products, advanced materials, and pharmaceuticals have made the preparation of C(sp^2^)–C(sp^2^) bond among the core interests of organic synthesis [1]. Transition metal-catalyzed cross-coupling reactions of aromatic compounds are useful synthetic routes to biaryl compounds [2,3,4,5,6,7]. By far, traditional transition metal-catalyzed biaryl cross-coupling reactions, which generally employ aryl halides and organometallics as coupling partners, have served as the most common methods for constructing biaryl unions (Figure 1a) [8,9]. Despite the large scope of reactions and opportunities opened by these traditional methodologies, the starting organometallic reagents are often not commercially available. Moreover, the amount of byproducts produced, whether in the synthesis of the organometallic reagent or during the coupling step itself, remains significant. Recently, there has been much interest in transition metal-catalyzed direct cross-coupling C–C bond formation of aromatic compounds with aryl halides as electrophilic partners involving the activation of normally unreactive aromatic C–H bond, in terms of synthesis efficiency and minimization of reaction steps waste (Figure 1b) [10,11,12]. Therefore, direct arylation reactions through cleavage of C–H bonds represent an environmentally and economically more attractive strategy.

The direct C–C bond formation reactions generally involve directing-group-assisted activation of sp^2^ C–H bonds of *ortho* aromatic C–H bonds. Many directing groups, such as acetyl, acetamino, carboxylic acid, oxazolyl, pyridyl, hydroxyl, imino, and cyano moieties, have been used for C–H bond activation [13,14,15,16,17,18,19,20,21,22,23,24]. The first example of a functional group assisted ruthenium-catalyzed C–H bond functionalization was reported by Lewis in 1986 [25]. Further pioneering work by Murai, Kakiuchi and Chatani on ruthenium-catalyzed hydroarylations showed the ability of ruthenium catalysts to activate C–H bonds selectively, via assistance of a coordinating functional group [26]. In 2001, the first ruthenium(II)-catalyzed chelation-assisted direct arylation with aryl bromides was performed by Oi, Inoue and co-workers [27]. In addition, Prades and Peris also reported the acetate-assisted arylation of 2-phenylpyridine using *N*-heterocyclic carbene-ruthenium complexes [28].

Recently, different direct arylations catalysed by ruthenium-based complexes have been described by the groups of Ackermann [29,30,31], Dixneuf [32], Davies [33] and others [34,35,36,37,38]. Several ruthenium-catalyzed direct arylations were also reported by our group [39,40,41]. Most of these reported direct arylations were performed either in NMP (*N*-methyl-2-pyrrolidone) or toluene as solvent. However, the demand for a more environmentally friendly chemistry opens a new horizon for the extended application of “green solvents” in direct C–H bond functionalizations as well [42]. In this connection, several ruthenium-catalyzed direct arylations in water or diethylcarbonate (DEC) as green solvent using an additive were reported by Dixneuf’s group [43,44,45,46,47,48,49].

We now described the synthesis and characterization of new unsymmetrical benzimidazolium halides (**1a**–**e**) and five new ruthenium(II) complexes of the general formula [RuCl_2_(η,^6^η^1^–1-arylmethyl–3–butylbenzimidazol–2–ylidene)] (**2a**–**e**). All new compounds were characterized by ^1^H NMR, ^13^C NMR, FT-IR spectroscopy and elemental analysis techniques. The single crystal structure of the benzimidazole ruthenium complex **2b** was also determined. These new complexes with KOAc partner were evaluated as catalyst precursors for the direct mono orthoarylation of 2-phenylpyridine with (hetero)aryl chlorides in water, without the need of surfactant. 

## 2. Results and Discussion

### 2.1. Preparation of Benzimidazolium Halides

**Scheme 1:**
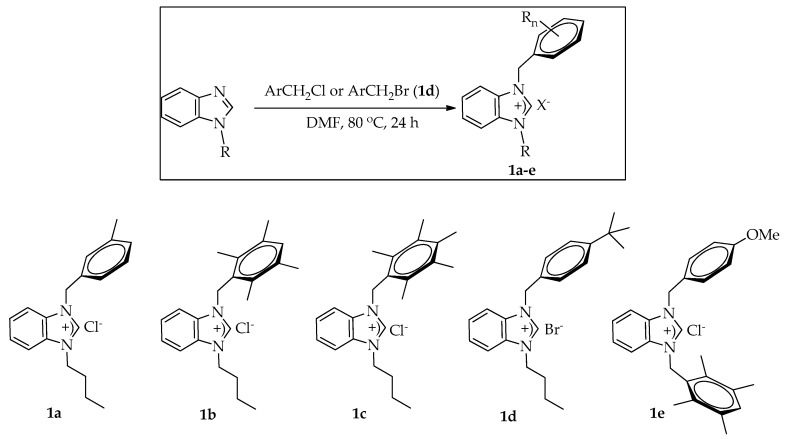
Synthesis of benzimidazolium halides, **1a**–**e**.

Benzimidazolium halides **1a**, **1b**, **1d** and **1e** were synthesized by reacting *N*-(alkyl)benzimidazole with alkyl halide in dimethylformamide (DMF) at 80 °C for 24 h. The benzimidazolium chloride 1c was obtained as we previously described [40]. These products were isolated as crystalline solids in high yields (78–87%). The benzimidazolium halides **1a**–**e** are air- and moisture-stable both in the solid state and in solution and are soluble in chlorinated solvents, ethyl alcohol and water (Scheme 1). The benzimidazolium halides were characterized using ^1^H NMR, ^13^C NMR, and FT-IR spectroscopy and gave satisfactory elemental analysis. The FT-IR data clearly indicated that the benzimidazolium halides **1a**–**e** exhibit a characteristic *ν*_(NCN)_ band typically between 1553–1560 cm^−1^. In the ^13^C NMR spectra of compounds **1a**–**e**, the characteristic peak of the imino carbon (N*C*HN) resonance was detected in the area *δ* 143.2–143.6 ppm. The ^1^H NMR spectra of compounds **1a**, **1b**, **1d** and **1e** further supported the assigned structures. The resonances for C(2) –*H* were observed as sharp singlets at *δ* 10.53–11.53 ppm and consistent with NMR values of other benzimidazolium halides [50].

### 2.2. Preparation of Ruthenium(II)-NHC Complexes Containing the η^6^,η^1^–NHC Mixed Chelating Ligand

**Scheme 2:**
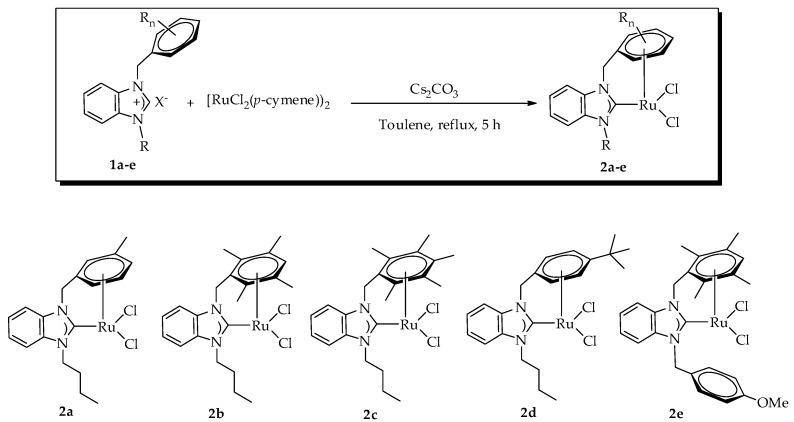
Synthesis of [Ru-Cl_2_(η^6^,η**^1^-**arene-CH_2_-NHC)] complexes, **2a**–**e**.

The carbene ligands formed by deprotonation of benzimidazolium salts **1a**–**e** using Cs_2_CO_3_ were reacted with [RuCl_2_(*p*–cymene)]_2_ in toluene at 110 °C for 5 h to give good yield of [RuCl_2_(η^6^,η^1^–1-arylmethyl–3–butylbenzimidazol-2-ylidene)] (Scheme 2). Selective crystallization by solvent diffusion technique (CH_2_Cl_2_/Hexane) allowed the formation of suitable orange-brown crystalline solids in yields of 70−82%. The air- and moisture-stable complexes **2a**–**e** are soluble in solvents such as dichloromethane, chloroform, toluene, and tetrahydrofuran. The ruthenium(II)–NHC complexes **2a**–**e** were characterized using ^1^H NMR, ^13^C NMR, and FT-IR spectroscopy and gave satisfactory elemental analysis. The FT-IR data clearly indicated that the exhibit a characteristic *ν*_(NCN)_ band typically within 1400–1407 cm^−1^. NMR analysis confirmed the loss of the *p*-cymene ligand and the coordination of the (arene-CH_2_-NHC)arene. ^13^C NMR chemical shifts provide a useful diagnostic tool for this type of metal carbene complex. The chemical shifts for the carbene carbon atom are located in *δ* 181.3–185.2 ppm range and are similar to those found in other ruthenium(II)–NHC complexes [51]. The analytical data are in good agreement with the compositions proposed for all the compounds we prepared, and are summarized in Table 1.

### 2.3. Single Crystal X-ray Diffraction and Structure Analysis of Complex ***2b***

**Figure 2:**
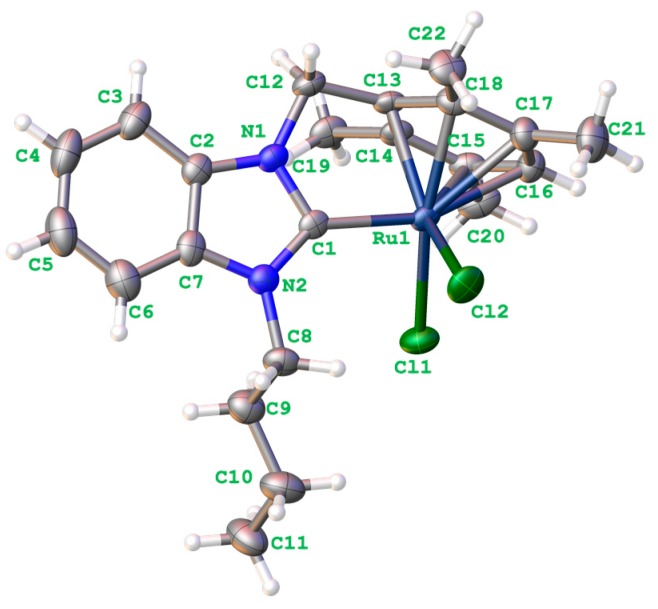
The molecular structure of compound **2b**. Displacement ellipsoids are drawn at the 50% probability level.

The ligand in compound **2b** has both benzimidazolylidene and arene rings, which are connected chelating via a CH_2_ bridge (Figure 2). Further details concerning data collection and refinements are given in the Supplementary Materials. This configuration leads to a system with very little apparent strain on coordinating the ruthenium atom. The complex is best described as containing an octahedrally coordinated Ru center with the arene residue occupying three sites, the remaining sites being occupied by an Ru–C(carbene) bond, derived from the benzimidazole ring, and two Ru–Cl bonds. The small steric demand of the benzimidazolylidene ligand is reflected in the C(1)–Ru(1)–C(13), C(1)–Ru(1)–Cl(1) and C(1)–Ru(1)–Cl(2) angles of 78.59(12), 92.94(9) and 89.64(8)°, respectively, a result that reflects the situation we found in related Ru complexes (Table 2) [52,53,54,55]. The atoms comprising the tetramethylbenzyl ring are coplanar, the mean deviation from their least squares plane being 0.017(2) Å. The five- and six-membered rings (N1/C1/N2/C7/C2) and (C2–C7) of the benzimidazolylidene group are almost co-planar with maximum deviations of 0.010(2) Å for N1 and 0.010(2) Å for C5, respectively. The dihedral angle between benzimidazolylidine and tetramethylbenzyl rings is 84.49 (16)°. The Ru-carbene distance, Ru(1)–C(1), of 2.040(3) Å, matches the literature data [52,53,54]. The Ru(1)–Cl(1) and Ru(1)–Cl(2) single bond lengths are 2.4267(9) and 2.4222(8) Å. The values of the geometrical parameters of compound **2b** agree with those reported for similar compounds [52,53,54].

### 2.4. Optimization Conditions of Direct Arylation of 2-Phenylpyridine with (hetero)Aryl Chlorides with Catalysts ***2***

The catalytic activity of the [RuCl_2_(η^6^,η^1^–1–arylmethyl–3-butylbenzimidazole–2–ylidene)] complexes **2a**–**e** for the activation of phenylpyridine towards (hetero)arylation of sp^2^ C–H bonds was investigated by the reaction of (hetero)aryl chloride and 2-phenylpyridine as a standard reaction. The reaction was carried out using 2-phenylpyridine, (hetero)aryl chloride and Cs_2_CO_3_ in the presence of complex **2c** as the catalyst in water with Cs**_2_**CO**_3_** as a base and a carboxylate RCO**_2_**K as additive. The results of varying the reaction parameters, including base, additive and reaction time are given in Table 3.

It has been shown by Dixneuf’s group [43,44,45,46,47,48,49] that carboxylates were efficient partners for ruthenium(II) C–H bond activation at room temperature, especially in water [44] The mechanism of the catalytic cycle showed that a carboxylate was crucial to deprotonate the C–H bond of aromatic systems [48,49]. Thus, pivalate and acetate additives were evaluated. When KOPiv was used as additive, the reaction gave low conversion of only 9% with Cs_2_CO_3_ after 5 h at 100 °C, and 100% (Table 3, entry 1). However, when K_2_CO_3_ and Na_2_CO_3_ were used as bases under similar conditions, no reaction took place (Table 3, entries 2 and 3). In the presence of KOAc as additive, the conversion was increased to 15% with Cs_2_CO_3_ after 5 h at 100 °C, to give mono-alkylated product, A, was also observed (Table 3, entry 4). When the reaction time was increased from 5 h to 10 h, the conversion was improved to 47% and 100% yield (Table 3, entry 5). Similarly, when the reaction time was increased from 10 to 20 h, the conversion was improved to 95% and 100% yield of product A was also observed (Table 2, entry 6). However, when the reaction time was increased from 20 to 24 h, no significant difference was observed in the conversion (Table 3, entry 7). These conditions (Table 3, entry 6) were selected for mono *ortho*-heteroarylation of phenylpyridine. As a result, the scope of the direct arylation of 2-phenylpyridine was investigated with 2-chlorothiophene applying our best experimental conditions (Table 3, entry 6). When 4-chlorotoluene was used as model coupling partner, the reaction gave full conversion (100% yield of mono-alkylated product A, was observed) with Cs_2_CO_3_ as base at different durations (5–20 h) at 100 °C (Table 3, entries 8–10). Eventually, the scope of the direct arylation of 2-phenylpyridine was investigated with 4-chlorotoluene applying our best experimental conditions (Table 3, entry 10). This shows that *ortho*-arylation is much easier than heteroarylation.

### 2.5. Direct Arylation of 2-Phenylpyridine with (Hetero)Aryl Chlorides

Based on this preliminary study, the scope of the direct arylation of 2-phenylpyridine was investigated with various (hetero)aryl chlorides applying our best experimental conditions to produce new potential bidentate ligands. With KOAc as additive, Cs_2_CO_3_ as base in water at 100 °C for different durations, ruthenium complexes **2a**–**e** were examined in the direct arylations of 2-phenylpyridine with six aryl chloride and thiophenyl chloride. In all of the reactions between different (hetero)aryl chlorides and 2-phenylpyridine, mono *ortho*-arylated product (A) was formed selectively. In addition, high conversion of *ortho*-arylated products was observed, and the selectivity was in high ratio towards to A. Although the other aryl chlorides showed good conversion in 5 h, chlorobenzene showed full conversion after 1 h. For example, when **2a** and **2b** complexes were used as catalyst, *ortho*-arylated product observed full conversion (selectivity of A 100%), (Table 4, entries 1 and 2). When the reaction of 2-phenylpyridine with 4-chlorotoluene was performed by compounds **2a**, **2b** and **2c**, *ortho*-arylated products (A and B) obtained full conversion (selectivity of A 100%), (Table 4, entries 6–8). When the reaction of 2-phenylpyridine with 4-chlorobenzaldehyde was performed by compounds **2a**–**e**, *ortho*-arylated products (A and B) obtained full conversion (Table 4, entries 11–15). When compound **2e** was used as catalyst, high conversion (>80%) was observed (selectivity of A >85%) (Table 4, entries 20 and 30). Heteroaryl derivative such as 2-chlorothiophene was also applicable to this direct arylation system (Table 4, entries 31–35). When compound **2c** was used as catalyst, *ortho*-arylated product was observed in conversion of 95% (selectivity of A 100%), (Table 4, entry 33). These results show that catalysts **2a**–**2b** were the best for monoarylation with simple phenyl and tolyl chlorides. For functional aryl chlorides ClC_6_H_4_Y, **2d** (Y = CHO), **2e** (Y = COMe and Y = CN), **2c** (thiophenyl chloride) appeared the most efficient.

## 3. Materials and Methods

### 3.1. General

All reactions for the preparation benzimidazolium halides (**1a**–**e**) and (arene)ruthenium(II)-(NHC)(Cl)_2_ complexes (**2a**–**e**) were carried out under argon in flame-dried glassware using standard Schlenk techniques. Chemicals and solvents were purchased from Sigma-Aldrich (Istanbul, Turkey) and Merck (Istanbul, Turkey). The solvents used were purified by distillation over the drying agents indicated and were transferred under Argon, Et_2_O (Na/K alloy), CH_2_Cl_2_ (P_4_O_10_), hexane, and toluene (Na). Microanalyses were performed by İnönü University Scientific and Technological Research Center (Malatya, Turkey). Melting points were determined in glass capillaries under air with an Electrothermal-9200 melting point apparatus (Cole-Parmer, Istanbul, Turkey). FT-IR spectra were recorded on ATR (Attenuated Total Reflection) unit in the range of 400–4000 cm^−1^ with Perkin Elmer Spectrum 100 Spectrophotometer (Istanbul, Turkey). Routine ^1^H NMR and ^13^C NMR spectra were recorded using a Bruker Avance AMX spectrometer (Ankara, Turkey) operating at 300 and 400 MHz for ^1^H NMR, and at 75 and 100 MHz for ^13^C NMR in CDCl_3_ with tetramethylsilane as an internal reference. Chemical shifts (*δ*) and coupling constants (*J*) are reported in ppm and in Hz, respectively. ^1^H NMR spectra are referenced to CDCl_3_ (*δ* = 7.26 ppm for CDCl_3_), ^13^C chemical shifts are reported relative to deuteriated solvent (*δ* = 77.16 ppm for CDCl_3_). All catalytic reactions were monitored on an Agilent 6890N Gas Chromatography (Ankara, Turkey) and Schimadzu 2010 Plus GC-MS system (Ankara, Turkey) by GC-FID (Flame Ionization Dedector) with a HP-5 column of 30 m length, 0.32 mm diameter and 0.25 μm film thickness. Column chromatography was performed using silica gel 60 (70–230 mesh).

### 3.2. General Procedure for the Preparation of Benzimidazolium Halides ***1a**–**e***

To a solution of 1-(*n*-butyl)benzimidazole (5.0 mmol) in DMF (5 mL) was added benzyl halide (5.0 mmol) and the resulting mixture was stirred at 80 °C for 5 h. After completion of the reaction, the solvent was removed by vacuum and diethylether (15 mL) was added to obtain a white crystalline solid, which was filtered off. The solid was washed with diethyl ether (3 × 10 mL) and dried under vacuum. The crude product was recrystallized from EtOH/Et_2_O mixture (1:2, *v*/*v*) and dried under vacuum. The ^1^H NMR and ^13^C NMR spectrums of new benzimidazolium halides (**1a**, **1b**, **1d** and **1e**) are available supporting information (Figures S1–S4).

*1-(3-Methylbenzyl)-3-(n-Butyl)Benzimidazolium Chloride,* **1a:** (1.228 g, yield 78%) ^1^H NMR (400 MHz, CDCl_3_, 25 °C): *δ* = 0.90 (t, ^3^*J* = 7.4 Hz, 3H, CH_2_CH_2_CH_2_C*H*_3_); 1.38 (hext, ^3^*J* = 7.5 Hz, 2H, CH_2_CH_2_C*H*_2_CH_3_); 1.97 (pent, ^3^*J* = 7.5 Hz, 2H, CH_2_C*H*_2_CH_2_CH_3_); 2.22 (s, 3H, CH_2_C_6_H_4_(C*H*_3_)-3); 4.57 (t, ^3^*J* = 7.4 Hz, 2H, C*H*_2_CH_2_CH_2_CH_3_); 5.79 (s, 2H, C*H*_2_C_6_H_4_(CH_3_)-3); 7.04 (d, ^3^*J* = 7.5 Hz, 1H, arom. CH, NC_6_*H*_4_N and CH_2_C_6_*H*_4_(CH_3_)-3); 7.13–7.21 (m, 3H, arom. CH, NC_6_*H*_4_N and CH_2_C_6_*H*_4_(CH_3_)-3); 7.47–7.57 (m, 3H, arom. CH, NC_6_*H*_4_N and CH_2_C_6_*H*_4_(CH_3_)-3); 7.67 (d, ^3^*J* = 8.2 Hz, 1H, arom. CH, NC_6_*H*_4_N and CH_2_C_6_*H*_4_(CH_3_)-3); 11.49 (s, 1H, NC*H*N) ppm. ^13^C NMR (100 MHz, CDCl_3_, 25 °C): *δ* = 13.5 (CH_2_CH_2_CH_2_*C*H_3_); 19.8 (CH_2_CH_2_*C*H_2_CH_3_); 21.3 (CH_2_C_6_H_4_(*C*H_3_)-3); 31.3 (CH_2_*C*H_2_CH_2_CH_3_); 47.5 (*C*H_2_CH_2_CH_2_CH_3_); 51.3 (*C*H_2_C_6_H_4_(CH_3_)-3); 113.1, 113.9, 125.3, 127.0, 127.1, 128.8, 129.1, 129.8, 131.2, 131.5, 132.9, 139.1 (arom. *C*, N*C*_6_H_4_N and CH_2_*C*_6_H_4_(CH_3_)-3); 143.2 (N*C*HN) ppm (Figure S1). Elemental analysis calcd (%) for C_19_H_23_ClN_2_ (Molar Mass (Mr) = 314.90): C 72.48, H 7.36, N 8.90; found (%): C 72.54, H 7.38, N 8.93.

*1-(2,3,5,6-Tetramethylbenzyl)-3-(n-Butyl)Benzimidazolium Chloride,* **1b:** (1.481 g, yield 83%) ^1^H NMR (300 MHz, CDCl_3_, 25 °C): *δ* = 0.87 (t, ^3^*J* = 7.0 Hz, 3H, CH_2_CH_2_CH_2_C*H*_3_); 1.33 (hext, ^3^*J* = 6.9 Hz, 2H, CH_2_CH_2_C*H*_2_CH_3_); 1.91 (pent, ^3^*J* = 6.5 Hz, 2H, CH_2_C*H*_2_CH_2_CH_3_); 2.16 (s, 12H, CH_2_C_6_H(C*H*_3_)_4_-2,3,5,6); 4.61 (t, ^3^*J* = 6.3 Hz, 2H, C*H*_2_CH_2_CH_2_CH_3_); 5.85 (s, 2H, C*H*_2_C_6_H(CH_3_)_4_-2,3,5,6); 6.98 (s, 1H, arom. CH, CH_2_C_6_*H*(CH_3_)_4_-2,3,5,6); 7.22 (d, ^3^*J* = 8.2 Hz, 1H, arom. CH of benzimidazole, NC_6_*H*_4_N); 7.40 (t, ^3^*J* = 7.7 Hz, 1H, arom. CH of benzimidazole, NC_6_*H*_4_N); 7.53 (t, ^3^*J* = 7.4 Hz, 1H, arom. CH of benzimidazole, NC_6_*H*_4_N); 7.69 (d, ^3^*J* = 8.0 Hz, 1H, arom. CH of benzimidazole, NC_6_*H*_4_N); 10.86 (s, 1H, NC*H*N) ppm. ^13^C NMR (75 MHz, CDCl_3_, 25 °C): *δ* = 13.5 (CH_2_CH_2_CH_2_*C*H_3_); 16.0 (CH_2_C_6_H(*C*H_3_)_4_-2,3,5,6); 19.7 (CH_2_CH_2_*C*H_2_CH_3_); 20.5 (CH_2_C_6_H(*C*H_3_)_4_-2,3,5,6); 31.3 (CH_2_*C*H_2_CH_2_CH_3_); 47.5 (*C*H_2_CH_2_CH_2_CH_3_); 47.8 (*C*H_2_C_6_H(CH_3_)_4_-2,3,5,6); 113.1, 113.8, 127.0, 127.2, 127.9, 131.4, 131.5, 133.4, 134.0, 134.9 (arom. *C*, N*C*_6_H_4_N and CH_2_*C*_6_H(CH_3_)_4_-2,3,5,6); 142.9 (N*C*HN) ppm (Figure S2). Elemental analysis calcd (%) for C_22_H_29_ClN_2_ (Mr = 356.90): C 74.03, H 8.19, N 7.85; found (%): C 74.00, H 8.16, N 7.81.

*1-(4-tert-Butylbenzyl)-3-(n-Butyl)Benzimidazolium Bromide,* **1d:** (1.605 g, yield 80%) ^1^H NMR (400 MHz, CDCl_3_, 25 °C): *δ* = 0.99 (t, ^3^*J* = 7.4 Hz, 3H, CH_2_CH_2_CH_2_C*H*_3_); 1.26 (s, 9H, CH_2_C_6_H_4_(C(C*H*_3_)_3_)-4); 1.48 (hext, ^3^*J* = 7.7 Hz, 2H, CH_2_CH_2_C*H*_2_CH_3_); 2.07 (pent, ^3^*J* = 7.6 Hz, 2H, CH_2_C*H*_2_CH_2_CH_3_); 4.64 (t, ^3^*J* = 7.4 Hz, 2H, C*H*_2_CH_2_CH_2_CH_3_); 5.88 (s, 2H, C*H*_2_C_6_H_4_(C(CH_3_)_3_)-4); 7.37–7.39 (m, 2H, arom. CH, NC_6_*H*_4_N and CH_2_C_6_*H*_4_(C(CH_3_)_3_)-4); 7.47–7.49 (m, 2H, arom. CH, NC_6_*H*_4_N and CH_2_C_6_*H*_4_(C(CH_3_)_3_)-4); 7.57–7.64 (m, 2H, arom. CH, NC_6_*H*_4_N and CH_2_C_6_*H*_4_(C(CH_3_)_3_)-4); 7.68–7.74 (m, 2H, arom. CH, NC_6_*H*_4_N and CH_2_C_6_*H*_4_(C(CH_3_)_3_)-4); 11.45 (s, 1H, NC*H*N) ppm. ^13^C NMR (100 MHz, CDCl_3_, 25 °C): *δ* = 13.5 (CH_2_CH_2_CH_2_*C*H_3_); 19.9 (CH_2_CH_2_*C*H_2_CH_3_); 31.2 (CH_2_C_6_H_4_(C(*C*H_3_)_3_)-4); 31.3 (CH_2_*C*H_2_CH_2_CH_3_); 34.7 (CH_2_C_6_H_4_(*C*(CH_3_)_3_)-4); 47.6 (*C*H_2_CH_2_CH_2_CH_3_); 51.1 (*C*H_2_C_6_H_4_(C(CH_3_)_3_)-4); 113.1, 113.9, 126.3, 127.1, 127.2, 128.2, 129.8, 131.3, 131.5, 152.4 (arom. *C*, N*C*_6_H_4_N and CH_2_*C*_6_H_4_(C(CH_3_)_3_)-4); 142.6 (N*C*HN) ppm (Figure S3). Elemental analysis calcd (%) for C_22_H_29_BrN_2_ (Mr = 401.40): C 65.83, H 7.28, N 6.98; found (%): C 65.75, H 7.29, N 6.95.

*1-(2,3,5,6-Tetramethylbenzyl)-3-(3-Methoxybenzyl)Benzimidazolium Chloride,* **1e:** (1.831 g, yield 87%) ^1^H NMR (300 MHz, CDCl_3_, 25 °C): *δ* = 2.25 and 2.26 (s, 12H, CH_2_C_6_H(C*H*_3_)_4_-2,3,5,6); 3.76 (s, 3H, CH_2_C_6_H_4_(OC*H*_3_)-4); 5.88 (s, 2H, C*H*_2_C_6_H_4_(OCH_3_)-4); 5.89 (s, 2H, C*H*_2_C_6_H(CH_3_)_4_-2,3,5,6); 6.86 (d, ^3^*J* = 8.6 Hz, 2H, arom. CH, NC_6_*H*_4_N and CH_2_C_6_*H*_4_(OCH_3_)-4); 7.08 (s, 1H, arom. CH, CH_2_C_6_*H*(CH_3_)_4_-2,3,5,6); 7.20 (d, ^3^*J* = 8.1 Hz, 1H, arom. CH, NC_6_*H*_4_N and CH_2_C_6_*H*_4_(OCH_3_)-4); 7.38–7.51 (m, 4H, arom. CH, NC_6_*H*_4_N and CH_2_C_6_*H*_4_(OCH_3_)-4); 7.61 (d, ^3^*J* = 8.1 Hz, 1H, arom. CH, NC_6_*H*_4_N and CH_2_C_6_*H*_4_(OCH_3_)-4); 11.53 (s, 1H, NC*H*N) ppm. ^13^C NMR (75 MHz, CDCl_3_, 25 °C): *δ* = 16.2 and 20.6 (CH_2_C_6_H(*C*H_3_)_4_-2,3,5,6); 47.9 (*C*H_2_C_6_H(CH_3_)_4_-2,3,5,6); 51.1 (*C*H_2_C_6_H_4_(OCH_3_)-4); 55.3 (CH_2_C_6_H_4_(O*C*H_3_)-4); 113.6, 113.8, 114.6, 114.7, 125.1, 126.9, 127.0, 127.7, 129.9, 131.6, 133.6, 134.1, 135.1, 160.1 (arom. *C*, N*C*_6_H_4_N, CH_2_*C*_6_H(CH_3_)_4_-2,3,5,6 and CH_2_*C*_6_H_4_(OCH_3_)-4); 143.6 (N*C*HN) ppm (Figure S4). Elemental analysis calcd (%) for C_26_H_29_ClN_2_O (Mr = 421.00): C 74.18, H 6.94, N 6.65; found (%): C 74.24, H 6.96, N 6.67.

### 3.3. General Procedure for the Preparation of Ruthenium(II)NHC Complexes ***2a**–**e***

A suspension of the benzimidazolium halide (1.10 mmol), Cs_2_CO_3_ (1.10 mmol) and [RuCl_2_(*p*-cymene)]_2_ (0.50 mmol) in degassed toluene (20 mL) was heated under reflux for 5 h. The reaction mixture was then filtered while hot, and the volume was reduced to about 10 mL before the addition of *n*-hexane (15 mL). The precipitate formed was crystallized from CH_2_Cl_2_/hexane mixture (1:3, *v*/*v*) to give red-brown crystals. The ^1^H NMR and ^13^C NMR spectrums of new ruthenium(II)-NHC complexes (**2a**–**e**) are available supporting information (Figures S5–S9).

*Dichloro-[1-(3-methylbenzyl)-3-(n-butyl)Benzimidazol-2-ylidene]ruthenium(II),* **2a**: (0.391 g, yield 87%) ^1^H NMR (300 MHz, CDCl_3_, 25 °C): *δ* = 0.95 (t, ^3^*J* = 7.4 Hz, 3H, CH_2_CH_2_CH_2_C*H*_3_); 1.45 (hext, ^3^*J* = 7.6 Hz, 2H, CH_2_CH_2_C*H*_2_CH_3_); 1.87 (pent, ^3^*J* = 7.8 Hz, 2H, CH_2_C*H*_2_CH_2_CH_3_); 2.23 (s, 3H, CH_2_C_6_H_4_(C*H*_3_)-3); 4.39–4.51 (m, 2H, C*H*_2_CH_2_CH_2_CH_3_); 4.89 (dd, *J* = 66.5, 11.6 Hz, 2H, C*H*_2_C_6_H_4_(CH_3_)-3); 5.07 (s, 1H, arom. CH, CH_2_C_6_*H*_4_(CH_3_)-3); 5.40 (d, ^3^*J* = 5.6 Hz, 1H, arom. CH, CH_2_C_6_*H*_4_(CH_3_)-3); 5.79 (d, ^3^*J* = 6.0 Hz, 1H, arom. CH, CH_2_C_6_*H*_4_(CH_3_)-3); 5.93 (t, ^3^*J* = 5.8 Hz, 1H, arom. CH, CH_2_C_6_*H*_4_(CH_3_)-3); 7.32–7.37 (m, 3H, arom. CH of benzimidazole, NC_6_*H*_4_N); 7.42–7.46 (m, 1H, arom. CH of benzimidazole, NC_6_*H*_4_N) ppm. ^13^C NMR (75 MHz, CDCl_3_, 25 °C): *δ* = 13.9 (CH_2_CH_2_CH_2_*C*H_3_); 19.0 (CH_2_CH_2_*C*H_2_CH_3_); 20.0 (CH_2_C_6_H_4_(*C*H_3_)-3); 32.3 (CH_2_*C*H_2_CH_2_CH_3_); 47.8 (*C*H_2_CH_2_CH_2_CH_3_); 50.8 (*C*H_2_C_6_H_4_(CH_3_)-3); 87.4, 99.8, 100.6, 109.7, 111.9, 114.4, 123.4, 124.0, 132.8, 134.7 (arom. *C*, N*C*_6_H_4_N and CH_2_*C*_6_H_4_(CH_3_)-3); 181.3 (Ru-*C_carbene_*) ppm (Figure S5). Elemental analysis calcd (%) for C_19_H_22_Cl_2_N_2_Ru (Mr = 450.40): C 50.67, H 4.92, N 6.22; found (%): C 50.74, H 4.95, N 6.26.

*Dichloro-[1-(2,3,5,6-tetramethylbenzyl)-3-(n-butyl)benzimidazol-2-ylidene]ruthenium(II),* **2b**: (0.393 g, yield 80%) ^1^H NMR (300 MHz, CDCl_3_, 25 °C): *δ* = 0.94 (t, ^3^*J* = 7.4 Hz, 3H, CH_2_CH_2_CH_2_C*H*_3_); 1.44 (hext, ^3^*J* = 7.5 Hz, 2H, CH_2_CH_2_C*H*_2_CH_3_); 1.85 (pent, ^3^*J* = 7.5 Hz, 2H, CH_2_C*H*_2_CH_2_CH_3_); 1.99 and 2.13 (s, 12H, CH_2_C_6_H(C*H*_3_)_4_-2,3,5,6); 4.42–4.48 (m, 2H, C*H*_2_CH_2_CH_2_CH_3_); 5.06 (s, 2H, C*H*_2_C_6_H(CH_3_)_4_-2,3,5,6); 5.54 (s, 1H, arom. CH, CH_2_C_6_*H*(CH_3_)_4_-2,3,5,6); 7.32–7.38 (m, 2H, arom. CH of benzimidazole, NC_6_*H*_4_N); 7.41–7.44 (m, 2H, arom. CH of benzimidazole, NC_6_*H*_4_N) ppm. ^13^C NMR (75 MHz, CDCl_3_, 25 °C): *δ* = 13.8 (CH_2_CH_2_CH_2_*C*H_3_); 13.9 and 18.3 (CH_2_C_6_H(*C*H_3_)_4_-2,3,5,6); 20.0 (CH_2_CH_2_*C*H_2_CH_3_); 32.3 (CH_2_*C*H_2_CH_2_CH_3_); 45.9 (*C*H_2_C_6_H(CH_3_)_4_-2,3,5,6); 47.7 (*C*H_2_CH_2_CH_2_CH_3_); 84.5, 90.7, 97.5, 109.6, 110.2, 111.7, 123.1, 123.5, 133.0, 135.0 (arom. *C*, N*C*_6_H_4_N and CH_2_*C*_6_H(CH_3_)_4_-2,3,5,6); 184.9 (Ru-*C_carbene_*) ppm (Figure S6). Elemental analysis calcd (%) for C_22_H_28_Cl_2_N_2_Ru (Mr = 492.40): C 53.66, H 5.73, N 5.69; found (%): C 53.64, H 5.72, N 5.68.

*Dichloro-[1-(2,3,4,5,6-Pentamethylbenzyl)-3-(n-Butyl)Benzimidazol-2-Ylidene]Ruthenium(II),* **2c**: (0.415 g, yield 82%) ^1^H NMR (300 MHz, CDCl_3_, 25 °C): *δ* = 0.94 (t, ^3^*J* = 7.4 Hz, 3H, CH_2_CH_2_CH_2_C*H*_3_); 1.44 (hext, ^3^*J* = 7.5 Hz, 2H, CH_2_CH_2_C*H*_2_CH_3_); 1.84 (pent, ^3^*J* = 7.5 Hz, 2H, CH_2_C*H*_2_CH_2_CH_3_); 2.03, 2.09 and 2.21 (s, 15H, CH_2_C_6_(C*H*_3_)_5_-2,3,4,5,6); 4.42–4.47 (m, 2H, C*H*_2_CH_2_CH_2_CH_3_); 5.05 (s, 2H, C*H*_2_C_6_(CH_3_)_5_-2,3,4,5,6); 7.29–7.38 (m, 2H, arom. CH of benzimidazole, NC_6_*H*_4_N); 7.39–7.42 (m, 2H, arom. CH of benzimidazole, NC_6_*H*_4_N) ppm. ^13^C NMR (75 MHz, CDCl_3_, 25 °C): *δ* = 13.9 (CH_2_CH_2_CH_2_*C*H_3_); 14.9, 15.0 and 15.7 (CH_2_C_6_(*C*H_3_)_5_-2,3,4,5,6); 20.0 (CH_2_CH_2_*C*H_2_CH_3_); 32.3 (CH_2_*C*H_2_CH_2_CH_3_); 46.4 (*C*H_2_C_6_(CH_3_)_5_-2,3,4,5,6); 47.6 (*C*H_2_CH_2_CH_2_CH_3_); 86.0, 94.5, 97.9, 107.9, 109.5, 111.5, 123.0, 123.4, 133.2, 135.0 (arom. *C*, N*C*_6_H_4_N and CH_2_*C*_6_(CH_3_)_5_-2,3,4,5,6); 186.0 (Ru-*C_carbene_*) ppm (Figure S7). Elemental analysis calcd (%) for C_23_H_30_Cl_2_N_2_Ru (Mr = 506.50): C 54.54, H 5.97, N 5.53; found (%): C 54.57, H 5.98, N 5.54.

*Dichloro-[1-(4-tert-butylbenzyl)-3-(n-butyl)benzimidazol-2-ylidene]ruthenium(II),* **2d**: (0.349 g, yield 71%) ^1^H NMR (300 MHz, CDCl_3_, 25 °C): *δ* = 0.97 (t, ^3^*J* = 7.1 Hz, 3H, CH_2_CH_2_CH_2_C*H*_3_); 1.26 (s, 9H, CH_2_C_6_H_4_(C(C*H*_3_)_3_)-4); 1.46 (hext, ^3^*J* = 6.4 Hz, 2H, CH_2_CH_2_C*H*_2_CH_3_); 1.93–1.95 (m, 2H, CH_2_C*H*_2_CH_2_CH_3_); 4.65 (m, 2H, C*H*_2_CH_2_CH_2_CH_3_); 5.40 (dd, J = 52.0, 14.7 Hz, 2H, C*H*_2_C_6_H_4_(C(CH_3_)_3_)-4); 5.44 (s, 1H, arom. CH, CH_2_C_6_*H*_4_(C(CH_3_)_3_)-4); 5.71–5.84 (m, 3H, arom. CH, CH_2_C_6_*H*_4_(C(CH_3_)_3_)-4); 7.32–7.58 (m, 4H, arom. CH of benzimidazole, NC_6_*H*_4_N) ppm. ^13^C NMR (75 MHz, CDCl_3_, 25 °C): *δ* = 13.7 (CH_2_CH_2_CH_2_*C*H_3_); 19.9 (CH_2_CH_2_*C*H_2_CH_3_); 31.2 (CH_2_C_6_H_4_(C(*C*H_3_)_3_)-4); 31.7 (CH_2_*C*H_2_CH_2_CH_3_); 34.6 (CH_2_C_6_H_4_(*C*(CH_3_)_3_)-4); 48.0 (*C*H_2_CH_2_CH_2_CH_3_); 52.0 (*C*H_2_C_6_H_4_(C(CH_3_)_3_)-4); 81.6, 96.5, 112.8, 113.9, 126.0, 126.3, 126.4, 128.3, 130.5, 131.4, 131.5, 151.7 (arom. *C*, N*C*_6_H_4_N and CH_2_*C*_6_H_4_(C(CH_3_)_3_)-4); 186.1 (Ru-*C_carbene_*) ppm (Figure S8). Elemental analysis calcd (%) for C_22_H_28_Cl_2_N_2_Ru (Mr = 492.40): C 53.66, H 5.73, N 5.69; found (%): C 53.74, H 5.78, N 5.76.

*Dichloro-[1-(2,3,5,6-tetramethylbenzyl)-3-(4-methoxybenzyl)benzimidazol-2-ylidene]ruthenium(II),* **2e**: (0.422 g, yield 76%) ^1^H NMR (300 MHz, CDCl_3_, 25 °C): *δ* = 2.04 and 2.16 (s, 12H, CH_2_C_6_H(C*H*_3_)_4_-2,3,5,6); 3.74 (s, 3H, CH_2_C_6_H_4_(OC*H*_3_)-4); 5.12 (s, 2H, C*H*_2_C_6_H(CH_3_)_4_-2,3,5,6); 5.59 (s, 1H, arom. CH CH_2_C_6_*H*(CH_3_)_4_-2,3,5,6); 5.68 (s, 2H, C*H*_2_C_6_H_4_(OCH_3_)-4); 6.77 (d, ^3^*J* = 8.7 Hz, 2H, arom. CH, NC_6_*H*_4_N and CH_2_C_6_*H*_4_(OCH_3_)-4); 6.99 (d, *J* = 8.1 Hz, 1H, arom. CH, NC_6_*H*_4_N and CH_2_C_6_*H*_4_(OCH_3_)-4); 7.06–7.12 (m, 1H, arom. CH, NC_6_*H*_4_N and CH_2_C_6_*H*_4_(OCH_3_)-4); 7.20–7.28 (m, 3H, arom. CH, NC_6_*H*_4_N and CH_2_C_6_*H*_4_(OCH_3_)-4); 7.42 (d, ^3^*J* = 8.0 Hz, 1H, arom. CH, NC_6_*H*_4_N and CH_2_C_6_*H*_4_(OCH_3_)-4) ppm. ^13^C NMR (75 MHz, CDCl_3_, 25 °C): *δ* = 13.9 and 18.3 (CH_2_C_6_H(*C*H_3_)_4_-2,3,5,6); 46.0 (*C*H_2_C_6_H(CH_3_)_4_-2,3,5,6); 52.3 (*C*H_2_C_6_H_4_(OCH_3_)-4); 55.2 (CH_2_C_6_H_4_(O*C*H_3_)-4); 84.4, 90.6, 98.0, 109.5, 110.6, 113.0, 113.6, 123.1, 123.5, 129.0, 129.3, 133.2, 135.0, 158.8 (arom. *C*, N*C*_6_H_4_N, CH_2_*C*_6_H(CH_3_)_4_-2,3,5,6 and CH_2_*C*_6_H_4_(OCH_3_)-4); 185.2 (Ru-*C_carbene_*) ppm (Figure S9). Elemental analysis calcd (%) for C_26_H_28_Cl_2_N_2_ORu (Mr = 556.50): C 56.12, H 5.07, N 5.03; found (%): C 56.16, H 5.06, N 5.03.

### 3.4. General Procedure for the Direct Catalytic Arylation of 2-Phenylpyridine with (Hetero)Aryl Chlorides

Each [RuCl_2_(η^6^,η**^1^**-arene-CH_2_-NHC)] complex (**2a**–**e**) (0.025 mmol) and KOAc (0.05 mmol) was stirred in water (2 mL) at room temperature for 1 h, and then 2-phenylpyridine (0.5 mmol), (hetero)aryl chloride (1.25 mmol) and Cs_2_CO_3_ (1.50 mmol) were added. The resulting mixture was stirred at 100 °C for different durations. After completion of the reaction, the reaction mixture was cooled to room temperature. Dichloromethane was added to this mixture and organic phase was extracted. The extracted organic phase was dried over MgSO_4_ and concentrated under vacuum. The remaining residue was purified by column chromatography on silica gel (pentane/diethylether mixture, 1:5 *v*/*v*). The *ortho*-arylated products A and B conversion and ratio were determined by GC and GC-MS analyses.

### 3.5. Single Crystal X-ray Diffraction and Structure Analysis

A crystal of compound **2b** with dimensions of 0.31 × 0.13 × 0.07 mm^3^ was mounted on the tip of a glass fiber using epoxy and placed on a Bruker SMART 1000 CCD (Atlanta, GA, USA) sealed tube diffractometer with graphite monochromated MoKα (0.71073 Å) radiation. Data were measured at 373(2) K using a series of combinations of phi and omega scans. Data collection, indexing and initial cell refinements were all carried out using SMART software [55]. Frame integration and final cell refinements were done using SAINT software [56]. The structure was solved using Direct methods and difference Fourier techniques (SHELXTL, V6.12) [57]. Hydrogen atoms were placed in their expected chemical positions using the HFIX command and were included in the final cycles of least squares with isotropic Uijs related to the atoms ridden upon. All non-hydrogen atoms were refined anisotropically. Structure solution, refinement, graphics and generation of publication materials were performed using the software SHELXTL, Version 6.12 [57].

## 4. Conclusions

The above described reactions demonstrate that water can be used as solvent for direct C–H bond functionalization. It is noteworthy that, in addition to its role as safe, clean, and recoverable solvent, water also plays the role of a positive catalyst partner, as in the direct selective mono(hetero)arylation of 2-phenylpyridine**.** We have synthesized and characterized new benzimidazolium halides (**1a**–**e**) and five new [RuCl_2_(η^6^,η**^1^**-arene-CH_2_-NHC)] complexes (**2a**–**e**). Complexes **2a**–**e** with KOAc partner showed good catalytic activity for the direct arylation of 2-phenylpyridine with (hetero)aryl chlorides in water. The catalytic activity of [RuCl_2_(η^6^,η**^1^**-arene-CH_2_-NHC)] for mono heteroarylation of heterocyclic arene (pyridine arene) has the potential to reach bidentate new ligands in the future. There is no serious doubt that this field of research will increase significantly in the future, hopefully making another step towards more environmentally friendly and energy/resources saving processes.

## Figures and Tables

**Table 1:** Physical and spectroscopic properties of new compounds **1** and **2**.

Compound	Formula	Isolated Yield (%)	M.p. (Metlting Point) (°C)	*v*(CN) (Frequency) (cm^−1^)	^1^H NMR H(2) (ppm)	^13^C NMR C(2) (ppm)
**1a**	C_19_H_23_ClN_2_	78	198–199	1558	11.49	143.2
**1b**	C_22_H_29_ClN_2_	83	176–177	1553	10.86	142.9
**1d**	C_22_H_29_BrN_2_	80	184–185	1560	11.45	142.6
**1e**	C_26_H_29_ClN_2_O	87	191–192	1557	11.53	143.6
**2a**	C_19_H_22_Cl_2_N_2_Ru	87	231–232	1401	*-*	181.3
**2b**	C_22_H_28_Cl_2_N_2_Ru	80	311–312	1400	*-*	184.9
**2c**	C_23_H_30_Cl_2_N_2_Ru	82	319–320	1406	*-*	186.0
**2d**	C_22_H_28_Cl_2_N_2_Ru	71	298–299	1403	*-*	186.1
**2e**	C_26_H_28_Cl_2_N_2_ORu	76	310–311	1407	*-*	185.2

**Table 2:** Selected bond lengths (Å), angles (°) and torsion angles (°) for compound **2b**.

Bond Lenght		Bond Angles		Torsion Angle	
C(1)–N(2)	1.360(4)	N(2)–C(1)–N(1)	105.2(3)	N(2)–C(1)–Ru(1)–Cl(2)	−53.0(3)
C(1)–N(1)	1.361(4)	N(2)–C(1)–Ru(1)	138.3(2)	N(1)–C(1)–Ru(1)–Cl(2)	129.7(2)
C(1)–Ru(1)	2.040(3)	N(1)–C(1)–Ru(1)	116.5(2)	N(2)–C(1)–Ru(1)–Cl(1)	36.1(3)
C(2)–N(1)	1.386(4)	N(1)–C(12)–C(13)	106.3(2)	N(1)–C(1)–Ru(1)–Cl(1)	−141.1(2)
C(2)–C(7)	1.394(5)	C(14)–C(13)–Ru(1)	73.54(17)	N(2)–C(1)–N(1)–C(12)	176.2(3)
C(7)–N(2)	1.393(4)	C(18)–C(13)–Ru(1)	73.77(18)	Ru(1)–C(1)–N(1)–C(2)	177.82(19)
C(8)–N(2)	1.466(4)	C(12)–C(13)–Ru(1)	116.3(2)		
C(12)–N(1)	1.466(4)	C(1)–N(1)–C(2)	111.6(3)		
C(12)–C(13)	1.517(4)	C(1)–N(1)–C(12)	121.5(3)		
C(13)–Ru(1)	2.109(3)	C(2)–N(1)–C(12)	126.7(3)		
C(14)–Ru(1)	2.190(3)	C(1)–N(2)–C(7)	111.1(3)		
C(15)–C(16)	1.426(5)	C(1)–N(2)–C(8)	124.5(3)		
C(15)–C(20)	1.513(4)	C(7)–N(2)–C(8)	124.3(3)		
C(15)–Ru(1)	2.241(3)	–––			
C(16)–Ru(1)	2.274(3)	–––			
C(17)–Ru(1)	2.267(3)	–––			
C(18)–Ru(1)	2.196(3)	–––			
Cl(1)–Ru(1)	2.4267(9)	–––			
Cl(2)–Ru(1)	2.4222(8)	–––			

**Table 3:**
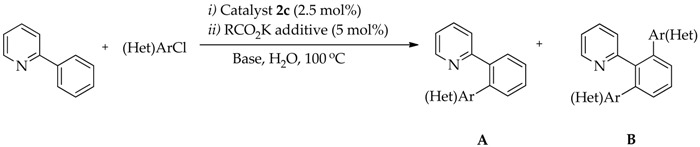
Arylation of 2-phenylpyridine with (hetero)aryl chlorides: A search for optimal catalytic conditions ^a^.

Entry	(Het)ArCl	Additive	Base	Time (h)	Conversion (%)	Yield (%)	
						A	B
1	2-Chlorothiophene	KOPiv	Cs_2_CO_3_	5	9	100	*-*
2	2-Chlorothiophene	KOPiv	K_2_CO_3_	5	-	-	*-*
3	2-Chlorothiophene	KOPiv	Na_2_CO_3_	5	-	-	*-*
4	2-Chlorothiophene	KOAc	Cs_2_CO_3_	5	15	100	*-*
5	2-Chlorothiophene	KOAc	Cs_2_CO_3_	10	47	100	*-*
**6**	**2-Chlorothiophene**	**KOAc**	**Cs_2_CO_3_**	**20**	**95**	**100**	*-*
7	2-Chlorothiophene	KOAc	Cs_2_CO_3_	24	97	100	*-*
**8**	**4-Chlorotoluene**	**KOAc**	**Cs_2_CO_3_**	**20**	**100**	**100**	*-*
**9**	**4-Chlorotoluene**	**KOAc**	**Cs_2_CO_3_**	**10**	**100**	**100**	*-*
10	4-Chlorotoluene	KOAc	Cs_2_CO_3_	5	100	100	*-*

^a^ Reaction conditions: Additive (0.05 mmol), ruthenium(II)-NHC complex, **2c** (0.025 mmol), H_2_O as solvent (2 mL), r.t. for 1 h, then 2-phenylpyridine (0.5 mmol), (hetero)aryl chloride (1.25 mmol), base (1.5 mmol). The conversions were based on the 2-phenylpyridine by GC (Gas chromatography) and GC-MS (Gas chromatography-Mass Spectrometry).

**Table 4:**
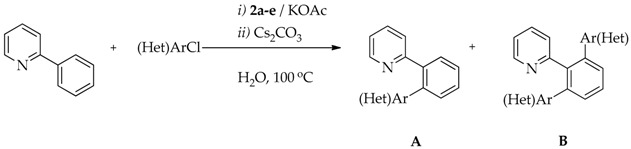
Direct arylation of 2-phenylpyridine with (hetero)aryl chlorides catalyzed by ruthenium(II)-NHC complexes **2a**–**e** ^a^.

Entry	Ru-NHC	(Het)ArCl	Time (h)	Conversion (%)	Yield (%)	
					A	B
1	**2a**	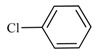	1	100	100	-
2	**2b**		1	100	100	-
3	**2c**		1	80	80	20
4	**2d**		1	90	90	10
5	**2e**		1	85	87	13
6	**2a**	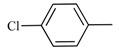	5	100	100	-
7	**2b**		5	100	100	-
8	**2c**		5	100	100	-
9	**2d**		5	100	88	12
10	**2e**		5	90	95	5
11	**2a**	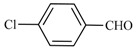	5	100	70	30
12	**2b**		5	100	80	20
13	**2c**		5	100	72	28
14	**2d**		5	100	92	8
15	**2e**		5	100	86	14
16	**2a**	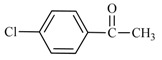	5	76	76	24
17	**2b**		5	72	72	28
18	**2c**		5	71	71	29
19	**2d**		5	70	70	30
**20**	**2e**		5	80	85	15
21	**2a**	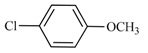	5	72	72	28
22	**2b**		5	77	77	23
23	**2c**		5	70	70	30
24	**2d**		5	79	79	21
25	**2e**		5	74	75	25
26	**2a**	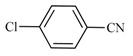	5	76	76	24
27	**2b**		5	71	71	29
28	**2c**		5	88	88	22
29	**2d**		5	85	85	15
30	**2e**		5	89	90	10
31	**2a**	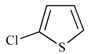	20	90	90	10
32	**2b**		20	88	95	5
33	**2c**		20	95	100	-
34	**2d**		20	89	97	3
35	**2e**		20	83	85	15

^a^ Reaction conditions: KOAc (0.05 mmol), ruthenium(II)-NHC complexes **2a**–**e** (0.025 mmol), H_2_O as solvent (2 mL), r.t. for 1 h, then 2-phenylpyridine (0.5 mmol), (Het)ArCl (1.25 mmol), Cs_2_CO_3_ (1.5 mmol).

## Supplementary Materials

Supplementary materials are available online. CCDC-1823696 contains the supplementary crystallographic data for this paper. These data can be obtained free of charge via www.ccdc.cam.ac.uk/data_request/cif, or by e-mailing data_request@ccdc.cam.ac.uk, or by contacting The Cambridge Crystallographic Data Centre, 12 Union Road, Cambridge CB2 1EZ, UK; Fax: +44(0)1223-336033.
