# Supplementary material for: Ruthenium(η6,η1-arene-CH2-NHC) Catalysts for Direct Arylation of 2-Phenylpyridine with (Hetero)Aryl Chlorides in Water
Source: Molecules. 2018 Mar 13;23(3):647. doi: 10.3390/molecules23030647 (PMC6017003; doi:10.3390/molecules23030647)
Supplement: Supplementary file 1 [file molecules-23-00647-s001.zip › supporting information/Supporting Information.docx]

| ***Contents*** | |
| --- | --- |
|  | |
| Characterising data of benzimidazolium chloride **1a** | **S1** |
| Characterising data of benzimidazolium chloride **1b** | **S2** |
| Characterising data of benzimidazolium bromide **1d** | **S3** |
| Characterising data of benzimidazolium chloride **1e** | **S4** |
| Characterising data of ruthenium(II)-NHC complex **2a** | **S5** |
| Characterising data of ruthenium(II)-NHC complex **2b** | **S6** |
| Characterising data of ruthenium(II)-NHC complex **2c** | **S7** |
| Characterising data of ruthenium(II)-NHC complex **2d** | **S8** |
| Characterising data of ruthenium(II)-NHC complex **2e** | **S9** |

*1-(3-Methylbenzyl)-3-(n-butyl)benzimidazolium Chloride,* ***1a***

**Figure S1.** ^1^H and ^13^C NMR spectrum of benzimidazolium chloride, **1a** (in CDCl_3_, 400 MHz).

*1-(2,3,5,6-Tetramethylbenzyl)-3-(n-butyl)benzimidazolium Chloride,* ***1b***

**Figure S2.** ^1^H and ^13^C NMR spectrum of benzimidazolium chloride, **1b** (in CDCl_3_, 300 MHz).

*1-(4-tert-Butylbenzyl)-3-(n-butyl)benzimidazolium Bromide,* ***1d***

**Figure S3.** ^1^H and ^13^C NMR spectrum of benzimidazolium bromide, **1d** (in CDCl_3_, 400 MHz).

*1-(2,3,5,6-Tetramethylbenzyl)-3-(3-methoxybenzyl)benzimidazolium Chloride,* ***1e***

**Figure S4.** ^1^H and ^13^C NMR spectrum of benzimidazolium chloride, **1e** (in CDCl_3_, 300 MHz)

*Dichloro-[1-(3-methylbenzyl)-3-(n-butyl)benzimidazol-2-ylidene]ruthenium(II),* ***2a***

**Figure S5.** ^1^H and ^13^C NMR spectrum of ruthenium(II)-NHC complex, **2a** (in CDCl_3_, 300 MHz).

*Dichloro-[1-(2,3,5,6-tetramethylbenzyl)-3-(n-butyl)benzimidazol-2-ylidene]ruthenium(II),* ***2b***

**Figure S6.** ^1^H and ^13^C NMR spectrum of ruthenium(II)-NHC complex, **2b** (in CDCl_3_, 300 MHz).

*Dichloro-[1-(2,3,4,5,6-pentamethylbenzyl)-3-(n-butyl)benzimidazol-2-ylidene]ruthenium(II),* ***2c***

**Figure S7.** ^1^H and ^13^C NMR spectrum of ruthenium(II)-NHC complex, **2c** (in CDCl_3_, 300 MHz).

*Dichloro-[1-(4-tert-butylbenzyl)-3-(n-butyl)benzimidazol-2-ylidene]ruthenium(II),* ***2d***

**Figure S8.** ^1^H and ^13^C NMR spectrum of ruthenium(II)-NHC complex, **2d** (in CDCl_3_, 300 MHz).

*Dichloro-[1-(2,3,5,6-tetramethylbenzyl)-3-(4-methoxybenzyl)benzimidazol-2-ylidene]ruthenium(II),* ***2e***

**Figure S9.** ^1^H and ^13^C NMR spectrum of ruthenium(II)-NHC complex, **2e** (in CDCl_3_, 300 MHz).
